# Subject-Specific Calculation of Left Atrial Appendage Blood-Borne Particle Residence Time Distribution in Atrial Fibrillation

**DOI:** 10.3389/fphys.2021.633135

**Published:** 2021-05-11

**Authors:** Soroosh Sanatkhani, Sotirios Nedios, Prahlad G. Menon, Andreas Bollmann, Gerhard Hindricks, Sanjeev G. Shroff

**Affiliations:** ^1^Department of Bioengineering, University of Pittsburgh, Pittsburgh, PA, United States; ^2^Department of Electrophysiology, Massachusetts General Hospital, Boston, MA, United States; ^3^Department of Electrophysiology, Heart Center, University of Leipzig, Leipzig, Germany; ^4^Department of Cardiology, Cardiovascular Research Institute Maastricht (CARIM), Maastricht University Medical Center, Maastricht, Netherlands

**Keywords:** computational fluid dynamics, mean residence time, 3D shape analysis, indices of stasis risk, passive tracer transport

## Abstract

Atrial fibrillation (AF) is the most common arrhythmia that leads to thrombus formation, mostly in the left atrial appendage (LAA). The current standard of stratifying stroke risk, based on the CHA_2_DS_2_-VASc score, does not consider LAA morphology, and the clinically accepted LAA morphology-based classification is highly subjective. The aim of this study was to determine whether LAA blood-borne particle residence time distribution and the proposed quantitative index of LAA 3D geometry can add independent information to the CHA_2_DS_2_-VASc score. Data were collected from 16 AF subjects. Subject-specific measurements included left atrial (LA) and LAA 3D geometry obtained by cardiac computed tomography, cardiac output, and heart rate. We quantified 3D LAA appearance in terms of a novel LAA *appearance complexity index* (LAA-*ACI*). We employed computational fluid dynamics analysis and a systems-based approach to quantify residence time distribution and associated calculated variable (LAA mean residence time, *t*_m_) in each subject. The LAA-*ACI* captured the subject-specific LAA 3D geometry in terms of a single number. LAA *t*_m_ varied significantly within a given LAA morphology as defined by the current subjective method and it was not simply a reflection of LAA geometry/appearance. In addition, LAA-*ACI* and LAA *t*_m_ varied significantly for a given CHA_2_DS_2_-VASc score, indicating that these two indices of stasis are not simply a reflection of the subjects' clinical status. We conclude that LAA-*ACI* and LAA *t*_m_ add independent information to the CHA_2_DS_2_-VASc score about stasis risk and thereby can potentially enhance its ability to stratify stroke risk in AF patients.

## Introduction

Atrial fibrillation (AF) is the most common arrhythmia, affecting three to six million US patients a year. This number is rapidly increasing with 12.1 million AF patients expected by 2030 (Virani et al., 2020). Due to the complex morphology of the left atrial appendage (LAA), as compared with the relatively smooth-walled left atrium (LA), the LAA is a favored location for thrombus formation (Reddy et al., 2013). These thrombi are known to cause stroke in AF patients. The CHA_2_DS_2_-VASc score is the most commonly used index for making clinical decisions regarding the management of AF patients. While this index is based on clinical data, it does not incorporate the role of LA–LAA geometry or local hemodynamics in the thromboembolic risk assessment.

**Figure 1:**
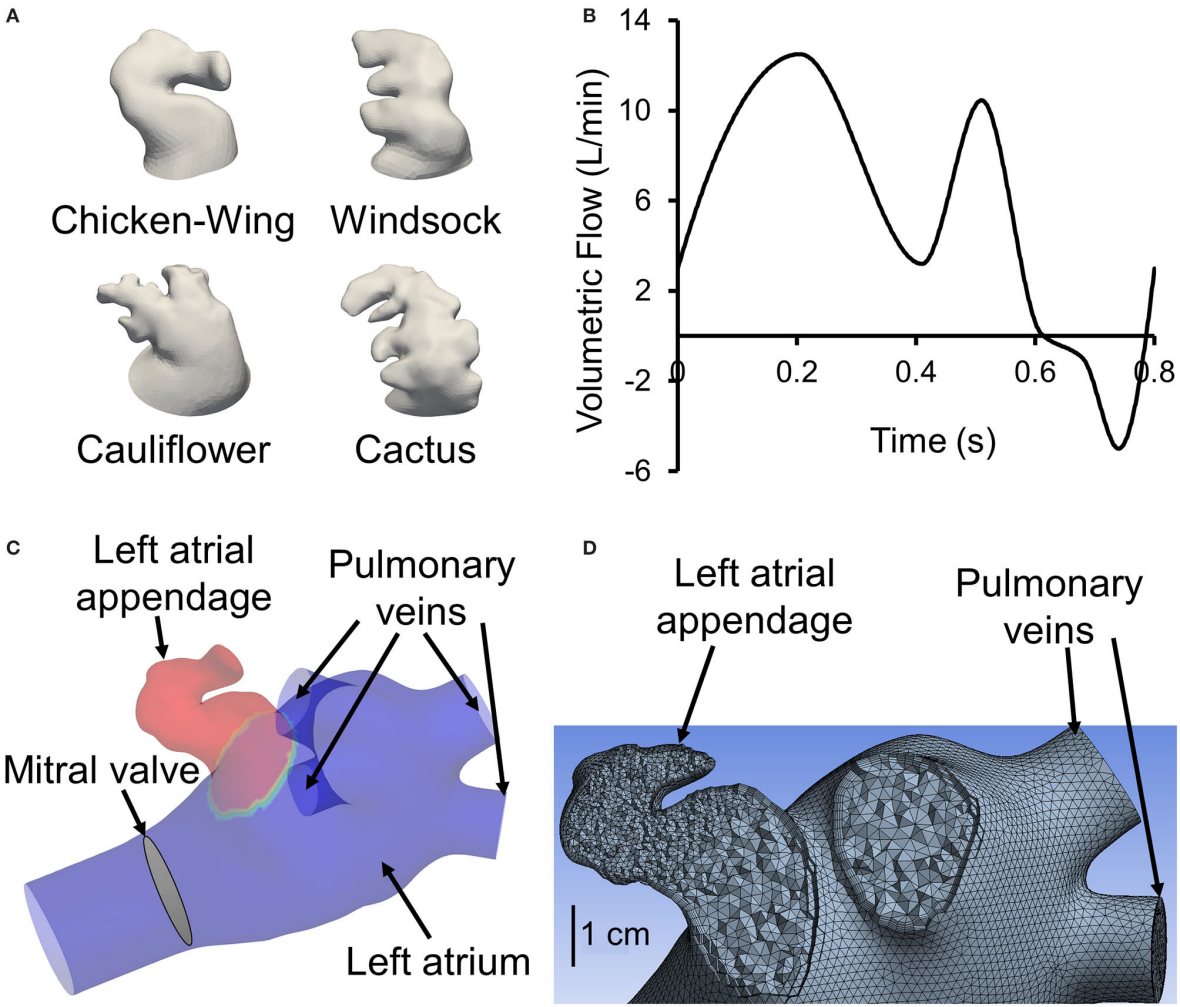
**(A)** One representative 3D reconstructed left atrial appendage (LAA) surface geometry from our cohort from each of the four categories proposed by Di Biase et al. (2012). **(B)** Generic flow waveform over one cardiac cycle used to generate subject-specific waveforms based on subject's cardiac output and heart rate (adapted from Smiseth et al., 1999). To avoid tracers exiting left atrial inlets, the waveform is shifted such that the backflow occurs at the end of the cardiac cycle around *t* = 0.6 s. **(C)** Sample geometry with LAA filled with tracer (colored red). All inlets (four pulmonary veins), the outlet (mitral valve), and the location of LAA are displayed. **(D)** A representative left atrium and LAA geometry meshed with tetrahedrons. A section through the appendage region shows a finer mesh inside the appendage. Furthermore, it shows the use of prismatic layers at the wall boundary.

The hypothesis that there is a correlation between the LAA morphology and stroke risk has been tested in several studies. Many indices have been examined in this context: LAA orifice diameter; number of branches and twigs; degree of coverage with fine structures (Ernst et al., 1995); LAA volume, depth, and number of lobes (Beinart et al., 2011); LAA takeoff from mitral valve (Nedios et al., 2014); and existence of a bend in LAA with an acute angle (Yaghi et al., 2020). Di Biase et al. (2012) categorized LAA shapes into four groups: chicken wing, windsock, cactus, and cauliflower shapes (Figure 1A). They concluded that patients with the chicken wing morphology are less likely to have a stroke. Although these results are promising, there is a large variability in stroke occurrence within a given LAA shape category (Khurram et al., 2013; Nedios et al., 2014; Sanatkhani and Menon, 2017; Yaghi et al., 2018). The subjective nature of LAA shape categorization may contribute to this variability. In an effort to objectify this, a recent study from our group has quantified LAA morphologies using principal component analysis (Sanatkhani and Menon, 2018). This approach uses the entire three-dimensional cardiac computed tomography (CCT) image, as opposed to isolated measurements of LAA dimensions, and therefore is more objective and comprehensive in quantifying LAA appearance.

Several surrogates of thrombus-promoting flow patterns have been used to relate blood flow in vascular structures (including LA and LAA) to probability of clot formation: wall shear stress, shear strain rate, time-averaged wall shear stress, oscillatory shear index (Koizumi et al., 2015), time-averaged velocity, mean resident time (Rayz et al., 2010), local residence time (Esmaily-Moghadam et al., 2013), residual virtual contrast agent (Otani et al., 2016; Bosi et al., 2018), and vortex structure (Masci et al., 2019a,b). The most realistic solution to simulate clot formation is to model the transport of blood cells (i.e., platelets, red blood cells, etc.) in a geometry. A Lagrangian approach can be used for this purpose (Bernsdorf et al., 2006), which requires tracking of a large number of particles and a very fine mesh to resolve the flow field for particle tracking, making it computationally expensive (Rayz et al., 2010). A Eulerian approach, which approximates particle tracking, has been used with reasonable success for quantifying indices correlated with thrombus formation (Rayz et al., 2010; Esmaily-Moghadam et al., 2013; Otani et al., 2016; Bosi et al., 2018; Sanatkhani et al., 2018; Masci et al., 2019b). However, the Eulerian approach has not been employed alongside a systems model approach to correlate the patient-specific LAA geometries with the residence time in LAA and consequently thrombus formation probability.

The present study has two aims: (1) to introduce a Eulerian approach for calculating the blood-borne particles residence time distribution (RTD) in patient-specific LAA and (2) to evaluate the correlation between RTD and known physical (e.g., LAA appearance and location) or clinical (e.g., CHA_2_DS_2_-VASc score) indices. This patient-specific morphology and hemodynamics-based approach of calculating RTD may add novel value to the current methodology of stratifying stroke risk and therefore enhance AF management-related decisions.

## Materials and Methods

### Data Acquisition

We performed CCT in a cohort of 16 AF subjects prior to an AF catheter ablation procedure. CCT images were obtained using a multidetector 64-row helical system (Brilliance 64, Philips, Netherlands). Image acquisition was ECG-gated when possible with the following parameters: 70–120 kV, 850 mA s, 0.6 mm beam collimation, 0.625–1.25 mm thickness, and 20–30 cm field-of-view. During an end-inspiratory breath-hold of 20 s and following a timing bolus-chase injection (20 ml at 5 ml s^−1^), 90 ml of an iodinated contrast medium (Ultravist 370, Bayer Vital, Cologne, Germany) was administered. Furthermore, subject-specific cardiac output and heart rate were measured. Heart rate and cardiac output data were used to scale (both the time and flow axes) a template LA inlet flow waveform (pulmonary vein flow) to generate a subject-specific LA inlet flow waveform (Figure 1B).

### Image Segmentation

Contrast-enhanced CCT DICOM images were cropped and then smoothed using a median filter with a kernel of 5 × 5 × 5. Images were segmented in ParaView (version 5.9.0, Kitware, Inc., Albuquerque, NM, USA) using the marching cubes method to create an iso-surface, representing the LA surface. The extracted surface included pulmonary veins (PV), the LA and LAA walls, and the mitral valve plane (excluding the valves themselves; Figure 1C). Extracted surfaces were smoothed out for computational fluid dynamics mesh (Figure 1D) by removing spikes and reducing noise (i.e., simplifying polygons) in Geomagic Studio (version 10, Geomagic, Inc., Research Triangle Park, NC, USA) and ANSYS SpaceClaim (version 2020 R2, ANSYS Inc., Canonsburg, PA, USA). A more detailed flowchart of LAA segmentation is presented in Sanatkhani and Menon (2018).

### LAA Appearance, LAA Location, and LAA–LA Size

**Figure 2:**
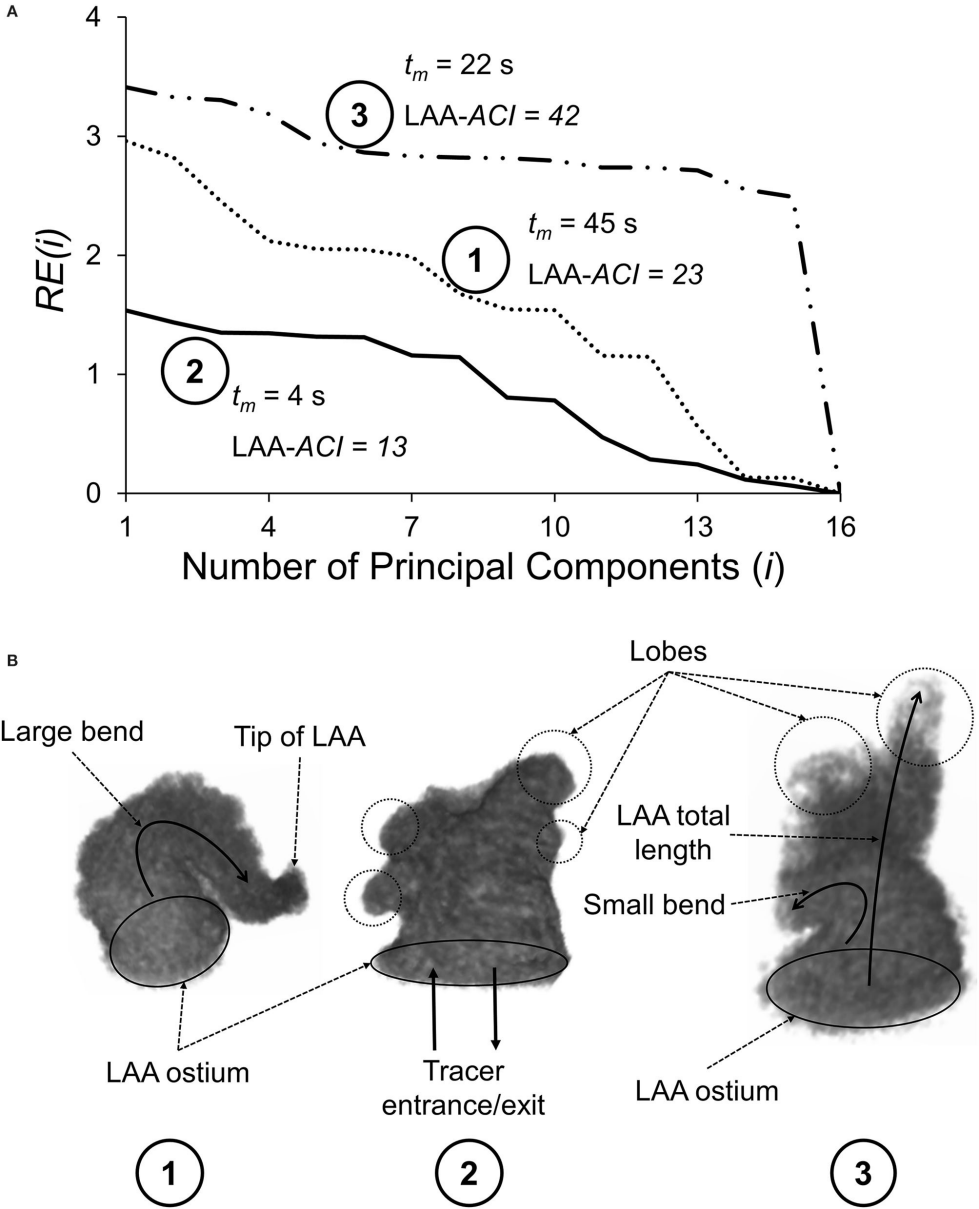
**(A)** Normalized residual error, *RE*(*i*), plotted as a function of the number of principal components (*i*) used to reconstruct the LAA appearance for three subjects. As *i* increases, *RE*(*i*) decreases, reaching a value of zero when *i* = 16, corresponding to a perfect reconstruction. LAA appearance complexity index (LAA-*ACI*) is defined as the area the area under the *RE*(*i*)–*i* curve; larger LAA-*ACI* corresponds to a more complex LAA appearance. **(B)** Geometrical features of these three LAAs, including LAA ostium, tip of the LAA, LAA lobes (shown by circles), and LAA centerline bend (shown by curved arrows). The rank ordering of these three LAAs based on the LAA-*ACI* (most complex to simplest) is subject #3, subject #1, and subject #2.

LAA appearance was quantified according to the methodology described in Sanatkhani and Menon (2018). Briefly, principal component analysis was used to generate the eigenvectors of the LAA appearance of each LAA in our study cohort. Next, we reconstructed each subject-specific LAA using a successively increasing number of principal components (PCs) and calculated a normalized residual error in appearance reconstruction for each step, *RE*(*i*), as follows (Equation 1):

(1) $$\begin{array}{l} RE\left(i\right)=\text{SQRT}\left[\frac{RSS\left(i\right)}{TSS}\right] \end{array}$$

where *RSS*(*i*) is the residual sum of squares for the *i*^th^ step (i.e., *i* PCs used in the reconstruction) and *TSS* is the total sum of squares. *RE*(*i*) decreases with increasing *i* because the more PCs we use to reconstruct an image, the more information is available to describe the details of the original image. Using all PCs would result in the original LAA image with zero residual error [*RE*(16) = 0]. We have defined the area under the curve of *RE*(*i*) vs. *i* as the LAA *appearance complexity index* (LAA-*ACI*); a larger area under the curve would correspond to a more complex appearance (e.g., see Figure 2A).

The LAA location with respect to the LA inlet and LA outlet was characterized by the distance from the center of the LAA ostium to the four inlets (i.e., pulmonary veins; *d*_PV1_, *d*_PV2_, *d*_PV3_, and *d*_PV4_) and the outlet (i.e., mitral valve; *d*_Mitral_). LAA volume (*V*_LAA_) and LA volume (*V*_LA_) were calculated using the 3D reconstructed CCT images. Distances are reported in centimeters and volumes in milliliters.

### Computational Fluid Dynamics

The prepared geometries were meshed in ANSYS Meshing. The maximum length for the tetrahedron edge was considered 3.5 mm for the whole geometry (including LA and LAA). Then, mesh was refined based on surface curvature to capture the topology. For instance, mesh at the tip of the LAA is finer than at the center of LA. Five prismatic layers at wall boundaries were used to resolve the boundary layer flow (Figure 1D). We used these settings for our course mesh. Next, we incrementally increased the number of elements until the changes in averaged wall shear stress in LAA were <5%. The number of mesh elements was chosen based on our mesh independency study. Based on the size and tortuosity of each subject, the number of mesh elements varied between 300,000 and 500,000 tetrahedrons, which was considered acceptable according to the literature (Otani et al., 2016; Aguado et al., 2019). LA and LAA walls were assumed to be rigid, impermeable, and with no-slip boundary conditions. Furthermore, for simplicity and lowering the computational costs, the mitral valve was assumed to be open throughout the simulation with both gauge pressure and velocity gradient set to zero. To prevent outlet backflow divergence, we extended the outlet in our geometries (Figure 1C) to develop a uniform with zero gradient velocity and pressure at the outlets. A velocity profile was prescribed at PV inlet boundaries. The PV waveform was generated by modifying a template normal waveform (Figure 1B) based on subject-specific cardiac output and heart rate. Blood was treated as an incompressible, Newtonian fluid with a density of ρ = 1, 060 kg m^−3^ and dynamic viscosity of μ = 0.00371 Pa s (Formaggia et al., 2009).

**Figure 3:**
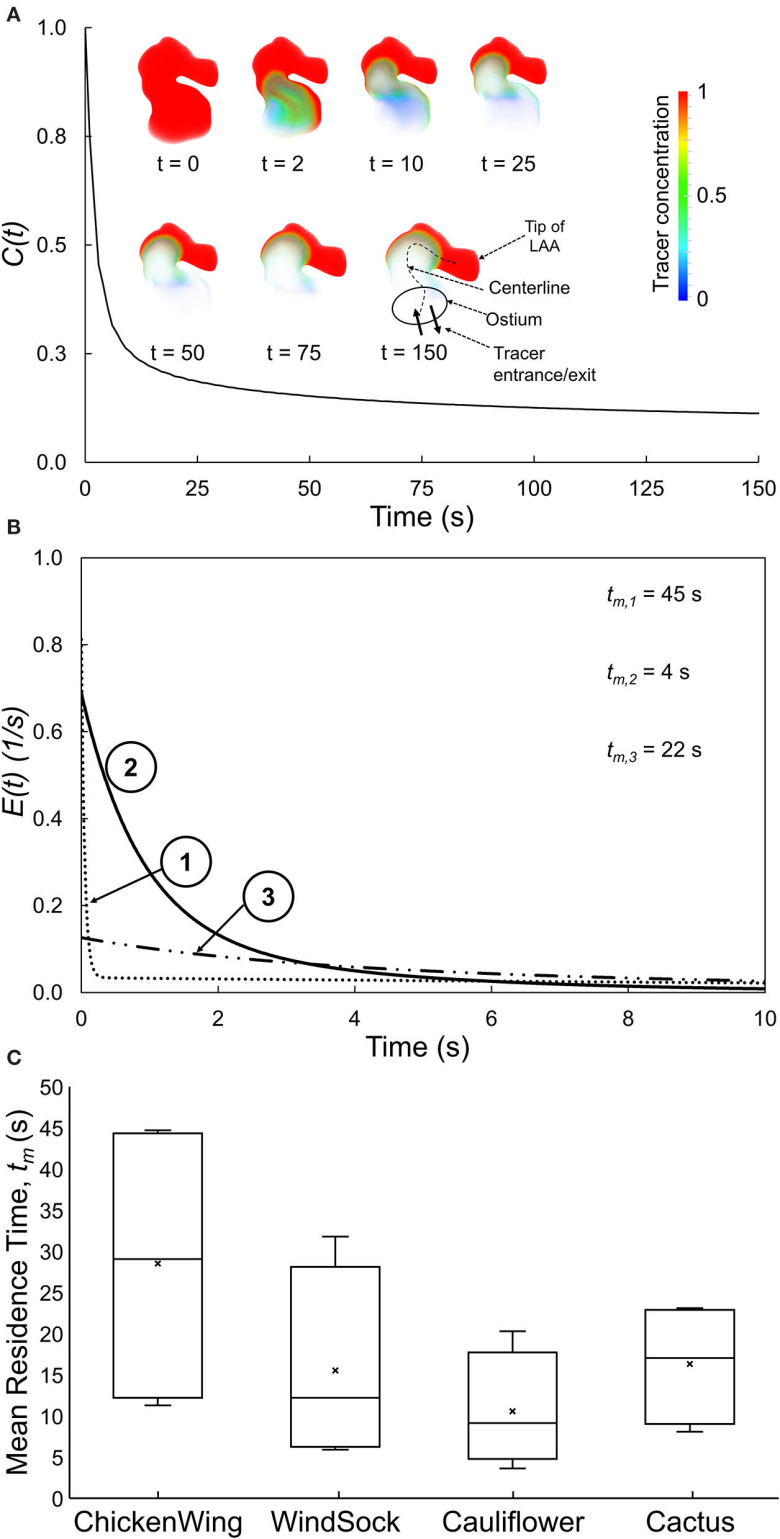
**(A)** Spatially averaged LAA tracer concentration, *C*(*t*), plotted as a function of time for a representative LAA. Inset: Tracer concentration contours for selected time, illustrating the tracer washout from most of the LAA, except for the tip of the LAA. **(B)** *E*(*t*), the residence time distribution function quantifying the normalized rate of tracer washout across the LAA ostium, as a function of time for three representative subjects. Data for the first 10 s are shown to highlight the early washout. **(C)** Box plots showing the mean residence time, *t*_m_, for each of the four traditional LAA shape groups. There is large variability in *t*_m_ within each traditional LAA shape group.

We first ran the fluid dynamics simulations until a hemodynamic steady-state was reached (after 25 cycles), as defined by the steady state of wall shear stress averaged over the LAA surface area of each subject. Thereafter, we performed simulations to analyze the transport of virtual tracer [i.e., passive scalar, representative of blood-borne particles (cells) that are neutrally buoyant in plasma] out of LAA. These tracer transport-related simulations were initialized with the LAA filled with the tracer concentration, *C*(*t*), of unity (representing an impulse filling of LAA with the tracer) (Figure 1C). Tracer advection was simulated using fluid dynamic analysis where the tracer concentration of each cell was calculated in the transport equation coupled with the momentum equations. The volumetric average of tracer concentration inside the LAA was recorded as *C*(*t*) for 150 s (Figure 3A). Based on the decay characteristics of *C*(*t*), we fitted a triple exponential model to *C*(*t*) that included an asymptotic term, *C*_∞_ (Equation A1).

The dynamics of the tracer clearance from LAA was quantified in terms of the RTD function, *E*(*t*) (Fogler, 2016):

(2) $$\begin{array}{l} \text{RTD Function}:\;E\left(t\right)=\frac{M\left(t\right)}{M_{total}} \end{array}$$

where *M*(*t*) is the outflow of tracer material (amount of tracer material per unit time) from LAA at the LAA ostium and *M*_total_ is the total amount of tracer that will leave the LAA over the period 0 to infinity. Thus, *E*(*t*), with the unit *per second*, represents the normalized outflow of tracer material from LAA at time *t*. As shown in the Appendix, we can rewrite Equation 2 in terms of the LAA tracer concentration, *C*(*t*), as follows:

(3) $$\begin{array}{l} E\left(t\right)=\frac{\left[C\left(t\right)-C\left(t+\Delta t\right)\right]}{\Delta t\left(1-C_{\infty }\right)} \end{array}$$

where Δ*t* and *C*_∞_ are the time increment used in the finite difference-based estimation of *M*(*t*) (Equation A3) and the asymptotic LAA concentration remaining in the LAA (Equation A1), respectively. Two measures of the propensity of particles to stay within the LAA were calculated: mean residence time, *t*_m_, which is the first moment of *E*(*t*) (Equation A6), and *C*_∞_ [*C*_∞_ = *C*(*t* → ∞), Equation A1]. A larger value for either of these two indices is expected to increase the clot formation risk.

The tracer transport was considered to happen with 0 m^2^ s^−1^ diffusivity, making the transport purely due to advection. We solved the transient transport equation for a scalar (i.e., tracer) using a laminar solver developed from IcoFoam and ScalarTransportFoam solvers in OpenFOAM (version 8, The OpenFOAM Foundation Ltd, Inc., UK). A time step of 500 μs was chosen based on a time-step independence study. We started with a 2 ms time step and decreased the value until the changes in averaged wall shear stress in LAA were <5%. The first-order implicit method was used for time discretization and second-order least-square scheme was used for pressure and velocity gradient discretization. First-order and second-order upwind schemes were used to discretize the divergence terms in the scalar transport equation and the convection term in Navier–Stokes equations, respectively. Pressure, velocity, and concentration tolerances were set to be 10^−7^, 10^−8^, and 10^−8^, respectively. For these simulations, 16 cores of Intel Zeon CPU with 2.7 GHz clock speed and 8 GB of RAM were used and the average execution time for each case was ~35 h. *C*(*t*) was extracted in ParaView from LAA and postprocessed in MATLAB® (version R2020b, MathWorks, Inc., Natick, MA).

### Statistical Analysis

Continuous variables are expressed as mean ± standard deviation. Correlations between variables were determined by Spearman rank correlation. The relationship between LAA *t*_m_ (dependent variable) and several independent (predictor) variables (e.g., LAA and LA morphological indices) was analyzed using backward multiple linear regression, which yielded the coefficients (β) of significant predictor variables and the respective 95% confidence interval (CI). The threshold for statistical significance, α, was set to 0.05. All statistical analyses were performed in SAS software (version 9.4, SAS Institute, Inc., NC, USA).

## Results

### Study Subject Characteristics

A total of 16 subjects with symptomatic AF (eight paroxysmal, eight persistent) were studied (four samples per four LAA shapes; 11 males). The average age, heart rate, and cardiac output were 60.3 ± 11.1 years (range: 33–78 years), 71 bpm (range: 42–100 bpm), and 4.2 L min^−1^ (range 2.7–6.0 L min^−1^), respectively. The range of CHA_2_DS_2_-VASc scores was 0 to 4 (mean = 2.2 ± 1.1).

### LAA Appearance Complexity Index (LAA-*ACI*)

The residual error for step *i, RE*(*i*), vs. *i* curves for three representative subjects is shown in Figure 2A, along with the geometry of the three LAAs (Figure 2B). Subject #3 requires significantly more PCs for accurate reconstruction and, therefore, has the largest LAA-*ACI* (i.e., area under the curve), indicating that this is the most complex appearance. The rank ordering based on the LAA-*ACI* is subject #3, subject #1, and subject #2. Interestingly, the rank ordering by the LAA mean residence time, *t*_m_, is subject #1, subject #3, and subject #2, suggesting that the LAA-*ACI* and *t*_m_ are not conveying the same information.

### Relating LAA RTD Function to Traditional LAA Shape Classification

The *C*(*t*) curve for a representative subject is shown in Figure 3A. The tracer washed out from the regions close to the LAA ostium after 2–10 s and tracer concentration continued to be high at the tip of the LAA even at the end of the simulation (*t* = 150 s).

The RTD function, *E*(*t*), for three representative subjects (same as those in Figure 2) is illustrated in Figure 3B, starting with the instant of the tracer introduction (*t* = 0) and until 10 s later. Subjects #1 and #3 started with lower initial normalized rate of tracer washout, *E*(*t*), across the LAA ostium, and this washout continued to remain lower as time progressed. Based on these *E*(*t*) curves and associated *t*_m_ values, the tracer exited from the LAA of subject #2 the fastest, followed by subjects #3 and subject #1 in that order.

The LAA appearance for each of the 16 subjects in the present study was classified into one of four groups by an electrophysiologist based on the study by Di Biase et al. (2012). Group data for LAA *t*_m_ (Figure 3C) indicated that LAA *t*_m_ had a large variability within each group, resulting in a significant overlap of this index of RTD function among the four LAA shape groups.

### Relating LAA Mean Residence Time to LAA Asymptotic Tracer Concentration

**Figure 4:**
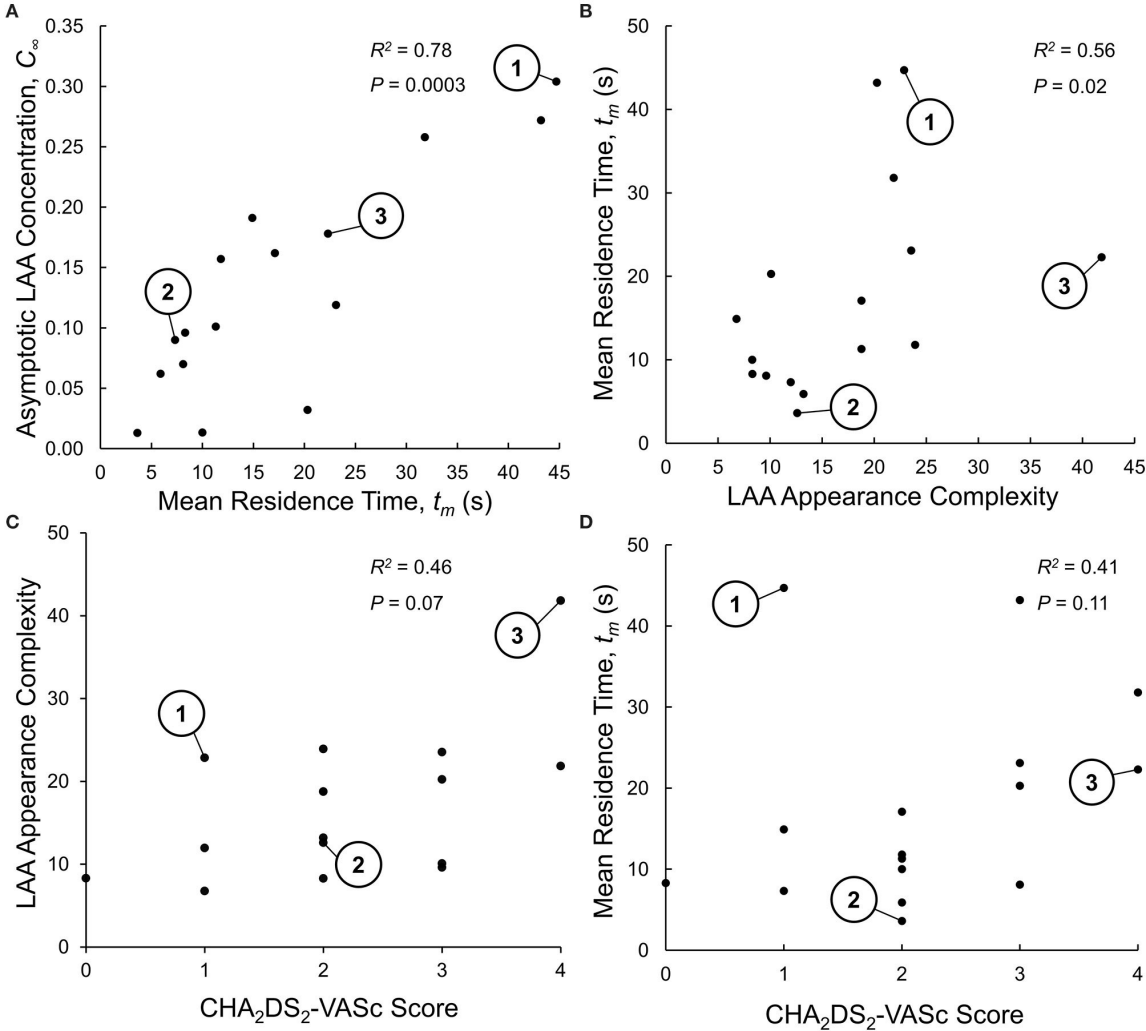
**(A)** Relationship between the two indices of LAA residence time distribution (RTD) function: mean residence time, *t*_m_, and aymptotic LAA concentration, *C*_∞_. The Spearman rank correlation analysis indicates that LAA *t*_m_ and *C*_∞_ are highly correlated, suggesting that only one of these indices is sufficent to characterizie the LAA RTD function. The three representative subjects shown in Figures 2, 3 are identified. **(B)** Relationship between LAA appearance complexity index (LAA-*ACI*) and LAA *t*_m_. The Spearman rank correlation analysis indicates a weak (although statistically significant) correlation, suggesting that these two variables do not provide the same infoamtion. **(C)** Relationship between LAA-*ACI* and CHA_2_DS_2_-VASc score, analyzed using Spearman rank correlation analysis, showing a weak and insignificant correlation. **(D)** Relationship between LAA *t*_m_ and CHA_2_DS_2_-VASc score, analyzed using Spearman rank correlation analysis, showing a weak and insignificant correlation.

The propensity of particles to stay within the LAA was characterized in terms of two indices: LAA mean residence time, *t*_m_, and LAA asymptotic tracer concentration, *C*_∞_. Spearman rank correlation analysis showed that there was a significant positive correlation between these two indices (*R*^2^ = 0.78, *P* = 0.0003; Figure 4A), suggesting that only one of these indices may be sufficient to characterize the propensity of particles to stay within LAA; we choose LAA mean residence time, *t*_m_.

### Relating LAA Mean Residence Time to LAA Morphology and Location

Given that the calculation of LAA *t*_m_ is computationally expensive, we wanted to examine whether LAA morphological indices (size, shape, and location) that are easier to measure can provide the same information. The Spearman rank correlation analysis indicated that only 56% of the variation in LAA *t*_m_ was explained by LAA-*ACI* (Figure 4B). We next carried out backward multiple linear regression analysis to investigate the relationship between LAA *t*_m_ (dependent variable) and eight independent variables: LAA-*ACI*, LAA location measures (*d*_PV1_, *d*_PV2_, *d*_PV3_, and *d*_PV4_, and *d*_*Mitral*_; see *LAA Appearance, LAA Location, and LAA–LA Size* section), and LA and LAA volumes (*V*_LA_ and *V*_LAA_). Only *d*_PV2_ and *V*_LAA_ were found to be significant predictor variables for *t*_m_. Although statistically significant, these two predictor variables could explain only 43% of the variation in *t*_m_. Thus, the LAA morphological indices do not provide the same information as *t*_m_.

### Relating LAA Mean Residence Time and LAA-*ACI* to CHA_2_DS_2_-VASc Score

Spearman rank correlation analysis was performed between LAA-*ACI* and CHA_2_DS_2_-VASc score (Figure 4C) as well as between LAA *t*_m_ and CHA_2_DS_2_-VASc score (Figure 4D). Both LAA-*ACI* (*R*^2^ = 0.46, *P* = 0.07; Figure 4C) and *t*_m_ (*R*^2^ = 0.41, *P* = 0.11; Figure 4D) were positively correlated with CHA_2_DS_2_-VASc score, but all these correlations were weak and did not reach statistical significance.

## Discussion

Lingering of blood cells inside the LAA could result in an elevated risk of thrombus formation and, consequently, stroke. In the present study, we quantify the propensity of blood cell lingering within the LAA in terms of the RTD function, *E*(*t*), and associated calculated variables (mean residence time of blood-borne particles in LAA, LAA *t*_m_, and asymptotic concentration remaining inside LAA, *C*_∞_). Both LAA and LA morphological features and spatially distributed hemodynamic milieu determine the LAA *t*_m_ and *C*_∞_. The key contributions of the present study are as follows: (1) the quantitation of the overall LAA appearance in terms of a novel index, the LAA-*ACI*; (2) the development of a Eulerian and systems-based approach for quantifying the LAA RTD function and associated calculated variable (LAA *t*_m_); (3) the observation that LAA-*ACI* and LAA location and size measures do not fully capture the information contained in LAA *t*_m_; and (4) the observation that the LAA-*ACI* and LAA *t*_m_ can add independent information to the CHA_2_DS_2_-VASc score and thereby potentially enhance its ability to stratify stroke risk in AF patients.

The idea that the complexity of LAA geometry plays an important role in stroke risk stratification in AF subjects is not new. As discussed in the *Introduction* section, many indices have been examined in this context, e.g., LAA orifice diameter; number of branches and twigs; degree of coverage with fine structure; LAA volume, depth, and number of lobes; and existence of a bend in LAA (Ernst et al., 1995; Beinart et al., 2011; Di Biase et al., 2012; Khurram et al., 2013; Nedios et al., 2014). While these are isolated features of the complex LAA geometry, our LAA-*ACI* utilizes the entire 3D dataset, and we believe that this integrated index incorporates the information provided by isolated measures. Considering the LAA-*ACI* values for three representative subjects illustrated in Figure 2, the LAA-*ACI* for subject #2 is the lowest, indicating that this subject has the simplest appearance. Subject #2 has a smoother wall and its total length (length of the LAA centerline from the ostium to the tip) is shorter compared with the other two. The appearance of subject #3 is the most complex, having several lobes (circled regions in Figure 2B). Furthermore, subject #3 has the longest length with a bend along its centerline. Although the LAA of subject #1 is smooth, its *ACI* falls in between the other two subjects because of the large bend along its centerline. Thus, our LAA-*ACI* is an objective and quantitative metric that characterizes the complexity of LAA appearance in a holistic way. The next question is whether this integrated index is superior from the perspective of improving the stroke risk stratification. We cannot answer this question at the present time; a longitudinal study will be necessary to address this question.

Other studies have used the classification paradigm of Di Biase et al. (2012) to characterize LAA shape. This is a very subjective approach and even experienced cardiologists may not always agree when classifying a LAA into specific shape categories. In addition, there is a large variability in stroke occurrence within a given LAA shape category (Khurram et al., 2013; Nedios et al., 2014; Sanatkhani and Menon, 2017; Yaghi et al., 2018). This is consistent with the large variability of *t*_m_ (Figure 3C) and LAA-*ACI* (data not shown) within each shape category and in LAA values that exist even within a given LAA shape category (Figure 3C). The intercategorical *t*_m_ (and LAA-*ACI*) variability may explain the differences in the stroke risk seen among subjects with similar overall LAA geometry. This variability underscores the importance of considering subject-specific LA and LAA morphologies in constructing a metric for stroke risk stratification in AF based on hemodynamics.

A systems-based approach was used to calculate *E*(*t*) in that it is the tracer washout response to an impulse injection of tracer in LAA. LAA *E*(*t*) curves are depicted in Figure 3B for the same three subjects as in Figure 2. Subject #2 and subject #1 had the highest and lowest starting points (i.e., value at *t* = 0), with subject #3 having an intermediate value. The lower values in subjects #1 and #3 are a consequence of the flow entering the LAA ostium that does not go all the way up to the LAA tip, resulting in a stagnant region at the tip. Subject #2 had the LAA *t*_m_. These data would predict that subject #1 has the highest risk of clot formation and subject #2 has the lowest risk.

There are other studies in the literature that have utilized more sophisticated computational fluid dynamics-based analysis to better mimic physiological conditions such as LA and LAA wall motion and more realistic LA outlet boundary conditions (Otani et al., 2016; Masci et al., 2019a). However, due to the very high computational costs associated with more complex computational fluid dynamic analyses, these studies were limited in terms of the number of subjects [five for Masci et al. (2019a) and four for Otani et al. (2016)] and the number of cardiac cycles analyzed (5 cycles). Based on our LAA mean residence time values (Figures 4A,B,D), we believe that it is necessary to perform the fluid dynamics simulation for at least 100 s. Our less sophisticated computational fluid dynamic analysis took about 35 h to complete an 150 s simulation (Intel Zeon CPU, 2.7 GHz, 16 cores). We believe that the more sophisticated analyses of Masci et al. (2019a) and Otani et al. (2016) will take at least several fold longer to perform an 150 s simulation, limiting their clinical application.

We have shown that the LAA *t*_m_ and LAA-*ACI* are weakly correlated (Figure 4B). This suggests that LAA *t*_m_, representing a holistic measure of subject-specific LA–LAA geometry features and hemodynamics, and LAA-*ACI* have a potential to contribute independent information. The observation that *d*_Mitral_ and *V*_LA_ were significant predictors of LAA *t*_m_ is in line with clinical data from a recent publication (Nedios et al., 2014) showing that a higher LAA takeoff, remote to the mitral valve plane, was associated with an increased thromboembolic risk.

We found a weak and insignificant correlation between the LAA-*ACI* and CHA_2_DS_2_-VASc score (Figure 4C) and between LAA *t*_m_ and CHA_2_DS_2_-VASc score (Figure 4D). In addition, LAA *t*_m_ varied significantly for a given CHA_2_DS_2_-VASc score (Figure 4D). Although subject #1 has a low CHA_2_DS_2_-VASc score (=1), this subject has the highest LAA *t*_m_ (Figure 4D). We would suggest that subject #1 has a high risk of stroke, despite the low CHA_2_DS_2_-VASc score. In contrast, there are two other subjects with CHA_2_DS_2_-VASc score of 1 and relatively low values of LAA *t*_m_ (Figure 4D); we would suggest that these subjects have a very low risk of stroke. These observations suggest that the hemodynamics-based index (i.e., LAA *t*_m_) and appearance indices (i.e., LAA-*ACI*) can add independent information to the CHA_2_DS_2_-VASc score.

## Limitations

Although we used subject-specific LA and LAA geometries, two concerns can be raised regarding the subject specificity of the fluid dynamics-based analysis. First, for LA inlet boundary conditions, a template (generic) LA inlet flow (pulmonary vein flow) waveform was used instead of a subject-specific waveform because patient-specific waveforms were not available for our cohort. However, because our preliminary study (Sanatkhani et al., 2020) showed that LAA *t*_m_ is significantly affected by mean LAA inlet flow (i.e., cardiac output), we scaled the template waveform of each subject to match the subject-specific cardiac output and heart rate. Future parametric studies will examine whether the temporal features of LAA inlet flow affect LAA *t*_m_. Second, for LA outlet boundary condition, the mitral valve was assumed to be open throughout the simulation, with both gauge pressure and velocity gradient set to zero. A better representation of LA outlet boundary condition would be in terms of left ventricular non-linear diastolic compliance and patient-specific left ventricular end-diastolic or end-systolic volume. These left ventricular diastolic compliance and volume data were not available for our cohort. We plan to conduct parametric studies to examine how left ventricular compliance and end-diastolic (or end-systolic) volume affect the calculated index, LAA *t*_m_.

We used the assumption of rigid LA and LAA walls. This assumption is an approximation for highly quivering LA and LAA in persistent AF patients, corresponding to the loss of coordinated contraction and consequent reduction in wall motion. A more realistic simulation with compliant LA and LAA walls would require the fluid–solid interaction approach for hemodynamic simulations, which significantly increases the complexity of the computational fluid dynamics analysis and associated computational cost. Alternatively, one can prescribe LA and LAA wall motions as boundary conditions; however, such wall motion data were not available for our cohort. It is recognized that some studies have used the prescribed wall motion in the computational fluid dynamics analysis of LA–LAA hemodynamics (Otani et al., 2016; Masci et al., 2019a). However, this approach requires additional data acquisition and significantly increases the computational costs, which may limit the clinical applicability. It has been shown that LAA thrombus formation is associated with both poor LAA contraction and LAA dilation (Pollick and Taylor, 1991). Therefore, the complete loss of wall motion may overestimate the risk of thrombus formation inside the LAA.

Furthermore, we assumed the blood to be a Newtonian fluid considering that LA domain is large and shear rate values are mostly in the range that allows for this assumption (mean shear strain rate, $\bar{\dot{\gamma }}=28.3\pm 45.9\;s^{-1}$). However, to better simulate the stasis regions inside the LAA, one might need to consider a non-Newtonian model. In our ongoing study, we are investigating the sensitivity of LAA *t*_m_ to various hematocrit using a non-Newtonian model in comparison with the Newtonian model.

Although our results indicate that LAA *t*_m_ and *ACI* provide additional information, a longitudinal study with a larger number of subjects will be needed to examine whether adding these indices to the CHA_2_DS_2_-VASc score can indeed enhance stroke prediction in AF.

## Summary and Conclusions

We have presented a novel index for quantifying the LAA geometry (LAA-*ACI*) and an approach for quantifying LAA residence time distribution and associated calculated variables using the subject-specific morphology, cardiac output, and heart rate with a hemodynamic model. Both the appearance index (i.e., LAA-*ACI*) and the hemodynamics-based index (i.e., LAA *t*_m_) add independent information to the CHA_2_DS_2_-VASc score about subject-specific stasis risk and thereby can potentially enhance its ability to stratify stroke risk in AF patients.

## Data Availability Statement

The raw data supporting the conclusions of this article will be made available by the authors, without undue reservation.

## Ethics Statement

Ethical review and approval was not required for the study on human participants in accordance with the local legislation and institutional requirements. Written informed consent to participate in this study was provided by the participants' legal guardian/next of kin.

## Author Contributions

SS, PM, and SGS designed the model and the computational framework and analyzed the data. SS performed the computations. SN, AB, and GH acquired the cardiac-CT data. SS, PM, and SGS wrote the manuscript with input from all authors. All authors contributed to the article and approved the submitted version.

## Conflict of Interest

The authors declare that the research was conducted in the absence of any commercial or financial relationships that could be construed as a potential conflict of interest.

## Abbreviations

AF: atrial fibrillation
LAA: left atrial appendage
LA: left atrium
RTD: residence time distribution
CCT: cardiac computed tomography
DICOM: digital imaging and communications in medicine
PV: pulmonary vein
PC: principal component
*RE*(*i*): residual error
*RSS*(*i*): residual sum of squares
*TSS*: total sum of squares
*ACI*: appearance complexity index
*d*_PV1_, *d*_PV2_, *d*_PV3_, and *d*_PV4_: distance from the center of the LAA ostium to the four inlets (i.e., pulmonary veins), respectively
*d*_Mitral_: distance from the center of the LAA ostium to the mitral valve
*V*_LA_: LA volume
*V*_LAA_: LAA volume
*C*′(*t*): concentration of tracer while exiting the LAA
*C*(*t*): tracer concentration inside the LAA
*E*(*t*): residence time distribution function
*t*_m_: mean residence time
*C*_∞_: asymptotic concentration remaining inside LAA
*Q*(*t*): volumetric flow exiting the LAA
β: multiple linear regression coefficient
CI: confidence interval
*M(t)*: outflow of tracer material from LAA at the LAA ostium
*M*_*total*_: total amount of tracer that will leave the LAA over the period 0 to infinity.

## Appendix

The computational fluid dynamics model yields LAA tracer concentration, *C*(*t*), every 1 s over the entire simulation period *t* = 0, 150 s (Figure 3A). The *C*(*t*) decay curve seems to have mutiple time constants and a non-zero asymptotic value (Figure 3A). Accordignly, a triple exponential model with non-zero asymptote was used to characterize the *C*(*t*) curve:

(A1) $$\begin{array}{l} C\left(t\right)=a_{1}e^{-b_{1}t}+a_{2}e^{-b_{2}t}+a_{3}e^{-b_{3}t}+C_{\infty } \end{array}$$

where *a*_1_, *a*_2_, *a*_3_, *b*_1_, *b*_2_, and *b*_3_ are the parameters to be estimated by fitting the triple exponential model (Equation A1) to the calculated *C*(*t*) data. Given that *C*(*t* = 0) = 1, *C*_∞_ = 1 − *a*_1_ − *a*_2_ − *a*_3_.

The dynamics of the tracer clearance from LAA was quantified in terms of the RTD function, *E*(*t*) (Fogler, 2016):

(A2) $$\begin{array}{l} \text{RTD Function}:\;E\left(t\right)=\frac{M\left(t\right)}{M_{total}} \end{array}$$

where *M*(*t*) is the outflow of tracer material (amount of tracer material per unit time) from LAA at the LAA ostium and *M*_total_ is the total amount of tracer that will leave the LAA over the period 0 to infinity. *M*(*t*) can be written in terms of *C*(*t*) using the finite difference approximation:

(A3) $$\begin{array}{l} M\left(t\right)=\frac{[C\left(t\right)-C\left(t+t)\right]V_{LAA}}{t} \end{array}$$

where Δ*t* and *V*_LAA_ are the time increment used in the finite difference-based estimation of *M*(*t*) and the LAA volume, respectively. To minimize errors in the finite difference-based estimation of *M*(*t*), we chose Δ*t* = 10^–6^ s.

Given that *C*(*t* = 0) = 1 and *C*(*t* = ∞) = *C*_∞_, *M*_total_ is given by:

(A4) $$\begin{array}{l} M_{total}=\left(1-C_{\infty }\right)V_{LAA} \end{array}$$

Substituting Equation A3 and Equation A4 in Equation A2, we get:

(A5) $$\begin{array}{l} E\left(t\right)=\frac{\left[C\left(t\right)-C\left(t+\Delta t\right)\right]}{\Delta t\left(1-C_{\infty }\right)} \end{array}$$

*E*(*t*)Δ*t* is the fraction of fluid exiting the LAA that has spent between time *t* and *t +* Δ*t* inside the LAA. Thus, the LAA mean residence time, *t*_m_, is given by the the first moment of *E*(*t*):

(A6) $$\begin{array}{l} t_{m}=\int_{0}^{\infty }tE\left(t\right)dt=\;\frac{1}{\Delta t\left(1-C_{\infty }\right)}\\ \;\int_{0}^{\infty }\left[tC\left(t\right)-tC\left(t+\Delta t\right)\right]dt \end{array}$$

It should be noted that since *C*(*t*) is comprised of exponetially decaying terms, $\int_{0}^{\infty }tC\left(t\right)\;dt$ is finite and the result can be written in terms of an analytical expression. For example, $\int_{0}^{\infty }te^{-at}dt\;=a^{-2}$. A custom program was developed in the MATLAB® (version R2020b, MathWorks, Inc., Natick, MA, USA) environment to estiamte the six parameters (*a*_1_, *a*_2_, *a*_3_, *b*_1_, *b*_2_, and *b*_3_) using a non-linear iterative optimization algorithm and perform subsequent data processing to calculate LAA mean residence time, *t*_m_.
